# Proteome and phosphoproteome reveal mechanisms of action of atorvastatin against esophageal squamous cell carcinoma

**DOI:** 10.18632/aging.102402

**Published:** 2019-11-07

**Authors:** Qiang Yuan, Christopher D. Dong, Yang Ge, Xinhuan Chen, Zhenzhen Li, Xin Li, Qiqi Lu, Feng Peng, Xiangyu Wu, Jimin Zhao, Kangdong Liu

**Affiliations:** 1 Department of Pathophysiology, School of Basic Medical Sciences, Zhengzhou University, Zhengzhou, Henan, China; 2 China-US Hormel (Henan) Cancer Institute, Zhengzhou, Henan, China; 3 Wartburg College, Waverly, IA 50677, USA; 4 School of Public Health, Shanghai Jiao Tong University School of Medicine, Shanghai, China; 5 Provincial Cooperative Innovation Center for Cancer Chemoprevention, Zhengzhou University, Zhengzhou, Henan, China; 6 Henan Provincial Key Laboratory of Esophageal Cancer, Zhengzhou, Henan, China; 7 Cancer Chemoprevention International Collaboration Laboratory, Zhengzhou, Henan, China

**Keywords:** atorvastatin, proteome, phosphoproteome, PDX, ESCC

## Abstract

Statins comprise a class of prescription drugs used for reducing cholesterol. Evidence has also showed that statins could reduce cancer incidence. However, the anti-tumor mechanism of statins has not been fully defined. Here, we found that atorvastatin inhibited proliferation of esophageal squamous cell carcinoma (ESCC) cells. The underlying mechanisms were explored by mass spectrometry. The proteome data revealed that atorvastatin inhibited the cAMP and Rap1 signal pathways, except for Ras signal pathway. Interestingly, phosphoproteome profiles suggested that ERK^T185/Y187^, CDK1^T14^, and BRAC1^S1189^ phosphorylation–mediated Th17 cell differentiation, Gap junction and the Platinum drug resistance pathway were down-regulated after atorvastatin treatment. The phosphorylation levels of ERK^T185/Y187^, CDK1^T14^ and BRAC1^S1189^ were confirmed by western blotting in KYSE150 cells. More importantly, atorvastatin suppresses ESCC tumor growth in PDX models. The molecular changes in tumor tissues were confirmed by immunohistochemistry. In conclusion, deep-proteome and phosphoproteome analysis reveal a comprehensive mechanism that contributes to atorvastatin’s anti-tumor effect.

## INTRODUCTION

Esophageal squamous cell carcinoma (ESCC) is an aggressive cancer which strikes a large number of people worldwide each year, especially in China [1]. Insufficient preventive measures and inadequate therapeutic techniques contribute to the poor five-year survival rate [2, 3]. Therefore, new drugs are urgently needed for ESCC prevention or therapy. Researchers have found that the statin family had an inhibitory effect in various cancers, such as colorectal cancer, breast cancer and melanoma [4, 5]. Atorvastatin, a member of the statin family, known as an inhibitor of 3-hydroxy-3-methylglutaryl coenzyme A (HMG-CoA) reductase in mevalonate pathway, has been used to reduce cholesterol level [6]. Beyond lowing cholesterol properties, increasing evidence has indicated that atorvastatin could suppress cell proliferation and induce cell apoptosis, through which atorvastatin inhibited carcinogenesis [7]. However, the detail mechanism of atorvastatin’s action against ESCC has not been fully elucidated.

In recent years, (phospho)proteomic base on mass spectrometry has been a comprehensive method to investigate alteration or modification of proteins [8]. We took advantage of high-resolution mass spectrometry (MS) to detect crucial molecular changes of ESCC cells after atorvastatin treatment. Proteomic study revealed that the Ras signal pathway was significantly down-regulated. More than that, the phosphoproteome indicated that Th17 cell differentiation, Gap junction, Platinum drug resistance, and the progesterone-mediated oocyte maturation pathway were dramatically down-regulated. These pathways are comprised of important proteins like ERK (MAPK1), CDK1 and BRCA1. The phosphorylation of ERK^T185/Y187^, CDK1^T14^ and BRCA1^S1189^ were significantly down-regulated. *In vivo*, atorvastatin suppressed ESCC tumor growth in patient-derived xenograft (PDX) models. These results indicated that atorvastatin inhibited the activation of key molecules, thereby rewiring tumor-dependent growth pathways. IHC analysis revealed that the molecular changes were consistent with the phosphoproteome.

## RESULTS

### Atorvastatin inhibits the proliferation of ESCC cells

**Figure 1:**
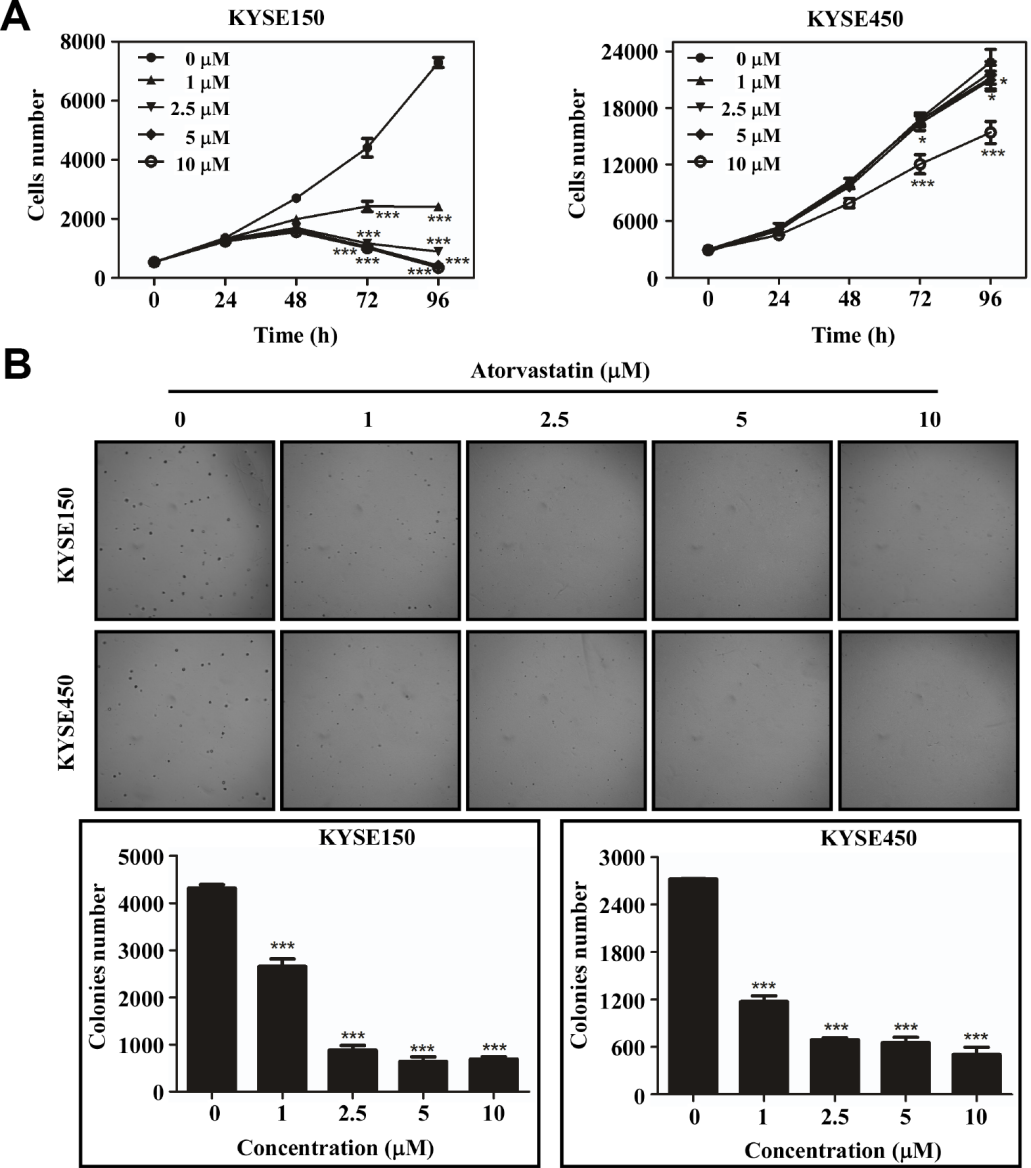
**Atorvastatin suppresses ESCC cells proliferation and anchor-independent cell growth.** (**A**) KYSE150 cells (left panel) and KYSE450 cells (right panel) were treated with atorvastatin (0, 1, 2.5, 5 and 10 μM). Cell number was counted at 0, 24, 48, 72, 96 h by analysis at IN Cell Analyzer 6000. Data are shown as means ± SD (* *P* < 0.05 *** *P* < 0.001 vs. untreated control, n ≥ 3). (**B**) Atorvastatin effectively inhibits anchorage-independent cell growth. KYSE150 and KYSE450 cells (8 ×10^3^/well) were exposed to different concentrations of atorvastatin with 1.25% Basal Medium Eagle agar containing 10% FBS and cultured for 8 days. Colonies were counted for analysis by IN Cell Analyzer 6000 soft agar program. Data are shown as means ± SD, (* *P* < 0.05, ** *P* < 0.01 *** *P* < 0.001 vs. untreated control, n ≥ 3).

To evaluate the effect of atorvastatin on ESCC growth, we treated KYSE150 and KYSE450 cells with different concentrations of atorvastatin. The results indicated that atorvastatin significantly attenuated growth of ESCC cells in a dose-dependent manner (Figure 1A) while having less effect against normal esophageal epithelial cell line SHEE (Supplementary Figure 1A). Next, the effect of atorvastatin on anchorage-independent growth of ESCC cells was evaluated by soft agar assay. Data suggested that atorvastatin treatment strongly inhibited anchorage-independent growth of KYSE150 and KYSE450 cells (Figure 1B). Therefore, atorvastatin inhibited the proliferation and anchorage-independent growth of ESCC cells.

### Mass spectrometry analysis based on proteome and phosphoproteome in atorvastatin-treated KYSE150 cells

**Figure 2:**
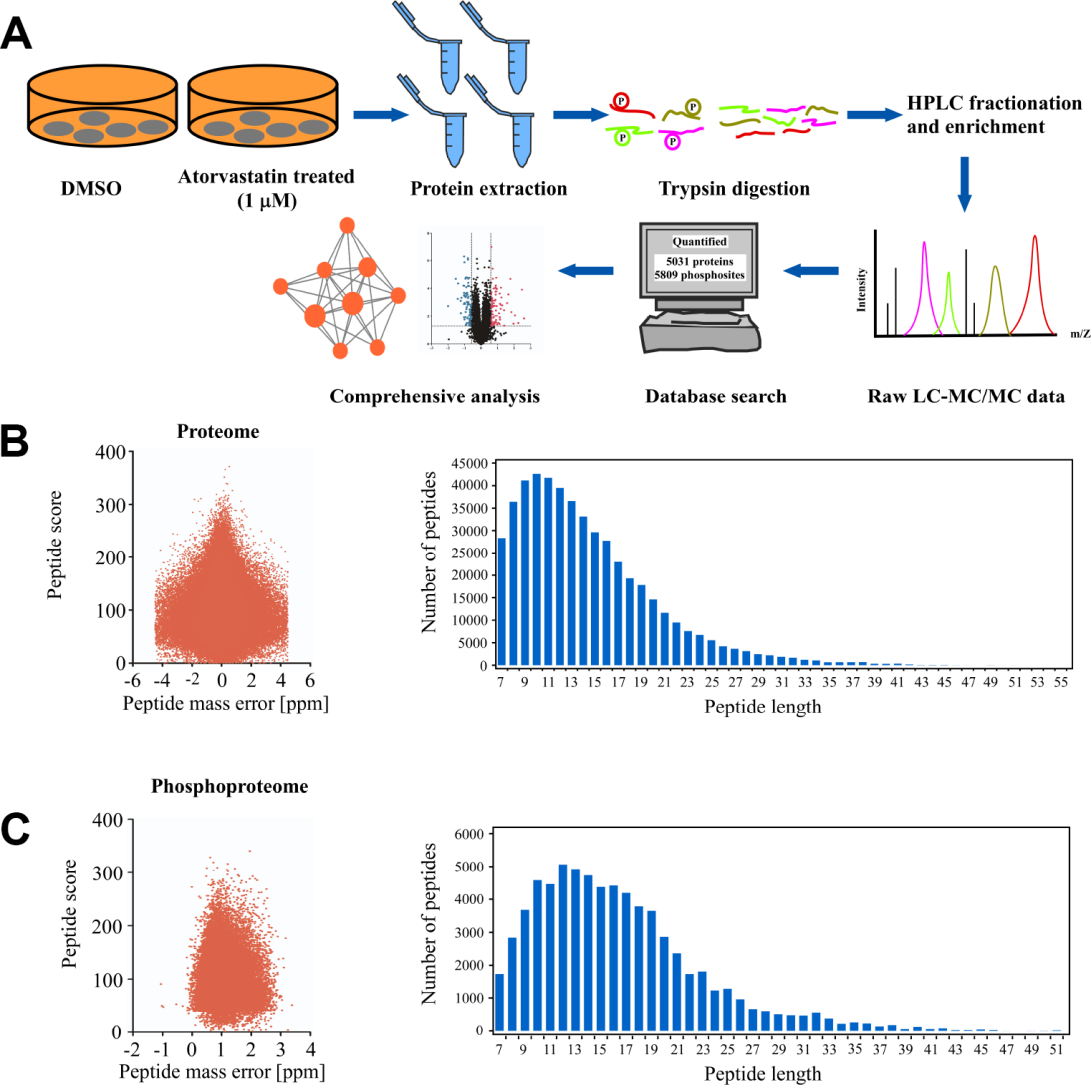
**Deep proteome and phosphoproteome analysis of atorvastatin-treated ESCC cells.** (**A**) general workflow of the experimental strategy to illustrate the processes in proteome, and phosphoproteome profiles of KYSE150 cells after 24 h of atorvastatin treatment (1 μM). (**B**) Mass error distribution map and peptide length distribution map of identified peptides in proteomics. (**C**) Mass error distribution map and peptide length distribution map of identified peptides in phosphoproteomics. The quality control report conforms to standard.

In order to comprehensively investigate the anti-mechanism of atorvastatin in ESCC, the KYSE150 cells were exposed to 1 μM atorvastatin or DMSO as control for 24 h. Subsequently, Mass spectrometry was performed. The whole experiment strategy was developed for quantitative (phosphor) proteomic profiles of atorvastatin-induced alteration with 3 biological replicates (Figure 2A). This approach quantified 5031 proteins (Supplementary Table 1) and 5809 phosphosites (Supplementary Table 2). The quality control report indicated that this test was in line with the standards (Figure 2B–2C).

### Proteomic analysis of the atorvastatin mode of action in ESCC cells

**Figure 3:**
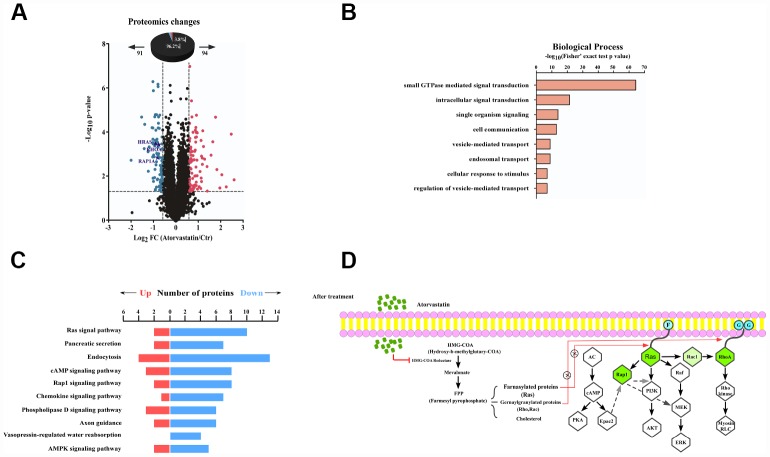
**Proteomic analysis reveals the anti-tumor mode of atorvastatin.** (**A**) volcano plot shows that 185 proteins changed significantly (*P* < 0.05 upon 24 h treatment with 1 μM atorvastatin). Blue dots represent down-regulated proteins, and red dots represent up-regulated proteins. (**B**) The picture shows biological processes of GO annotation. Bar chart represents biological processes enriched by altered proteins in response to atorvastatin treatment. The *P*-value (-log_10_) of each biological process is shown. (**C**) Pathways that are significantly down-altered in the proteome are shown on the left. The number of significantly regulated proteins in each pathway is shown in red and blue. (**D**) Proposed mechanisms underlying the action of atorvastatin's anti-tumor. Right shows that atorvastatin inhibits HMG-CoA reductase, and restrains the production of the Mevalonate pathway, which is necessary for Ras and RhoA. Left shows the changed pathway in response to atorvastatin treatment. Green dots are down-regulated proteins in the proteome.

We quantified and analyzed proteins from the raw database. A total of 185 proteins (3.6% of the 5031 proteins) significantly changed after atorvastatin treatment, among which 94 were up-regulated and 91 proteins were down-regulated respectively (Figure 3A). Interestingly, we found many down-regulated proteins correlated to the development of cancer. To thoroughly understand the role of the down-regulated proteins in the anti-tumor effect of atorvastatin, we annotated the function and characteristics of these proteins from the Gene Ontology (GO) and Kyoto Encyclopedia of Genes and Genomics (KEGG). GO analysis indicated the most two significantly changed biological processes were small GTPase mediated signal transduction and intracellular signal transduction. In addition to these observations, other functional categories are listed (Figure 3B). Consistent with GO analysis, the KEGG database showed that most drug-altered proteins were related to the Ras signal pathway (Figure 3C). The data also demonstrated that a large number of treatment-changed proteins were associated with the cAMP signal pathway and the Rap1 signal pathway (Figure 3C). In proteomic profiles, RhoA, Rap1, and Ras were strongly down-regulated, involved in these pathways mentioned above (Figure 3A). Multilayered proteomic results prompted us to depict a schematic of pathway interaction and protein alterations mediated by atorvastatin (Figure 3D). The picture showed that atorvastatin decreased the production of the mevalonate pathway by inhibiting HMG-CoA reductase, which is required for small G-protein activity, such as Ras and RhoA. In addition to these changes consistent with previous research, the proteome profile suggested the functional roles of altered proteins upon atorvastatin in the regulation of cAMP and Rap1 signal pathway.

### Phosphoproteomic profile was used to explore the anti-tumor mechanism through analyzing proteins altered upon atorvastatin treatment

**Figure 4:**
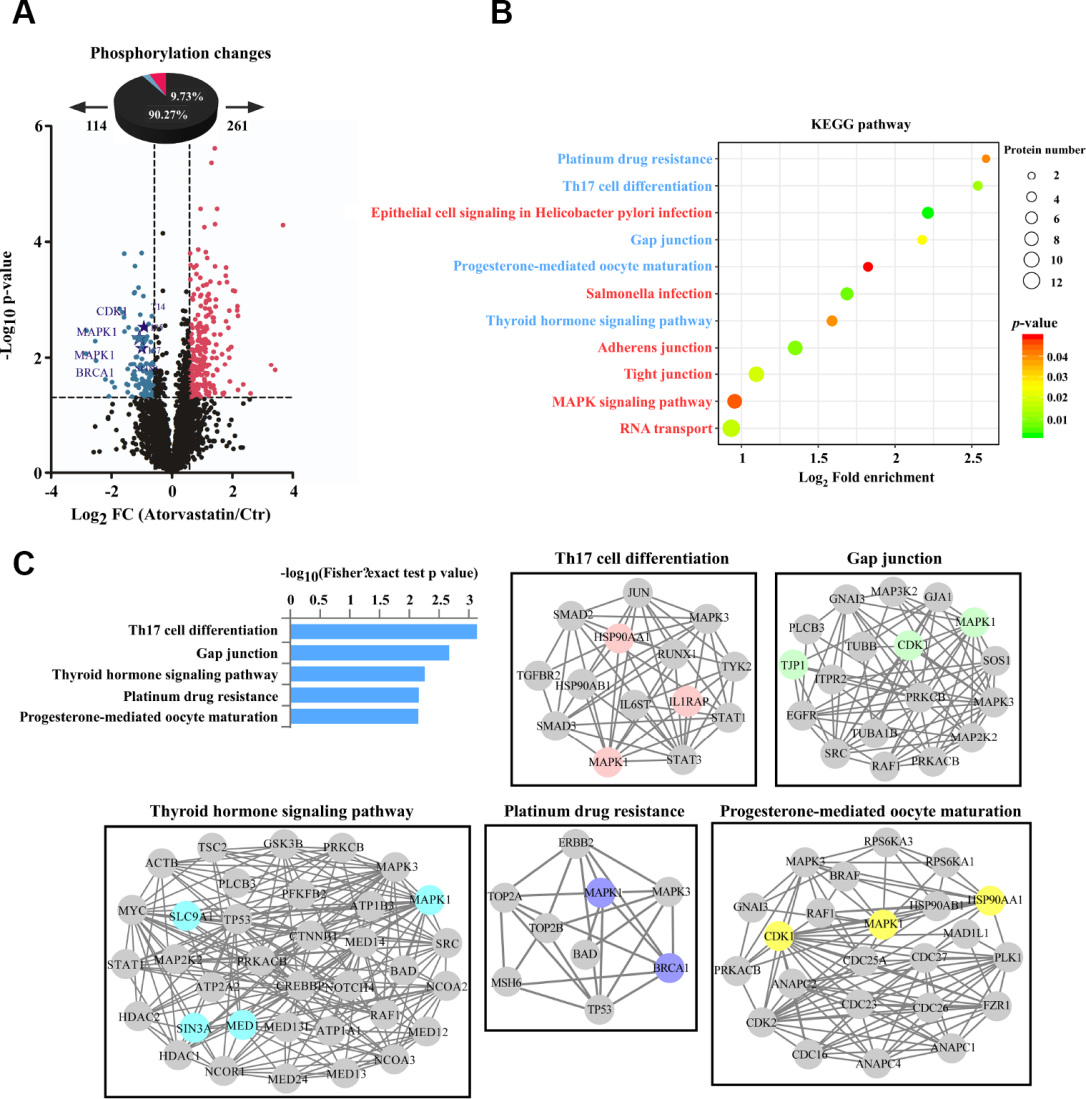
**Phosphorylation profiles reveal the anti-tumor mode of atorvastatin.** (**A**) Volcano plot shows that 375 phosphorylation sites changed significantly (*P* < 0.05 upon 24 h treatment with 1 μM atorvastatin). Blue dots represent down-regulated proteins, and red dots represent up-regulated proteins. (**B**) KEGG pathways that are significantly regulated (up or down) in phosphorylation are shown according to *P* -value, and the circle represents the number of mapping proteins. (**C**) According to the top 5 KEGG terms, these phosphoproteins whose sites were identified upon atorvastatin treatment are mapped to network by STRING database, in which the dot is protein. The colored nodes are significantly down-regulated phosphoproteins. The edges represent the STRING combined interaction score.

**Figure 5:**
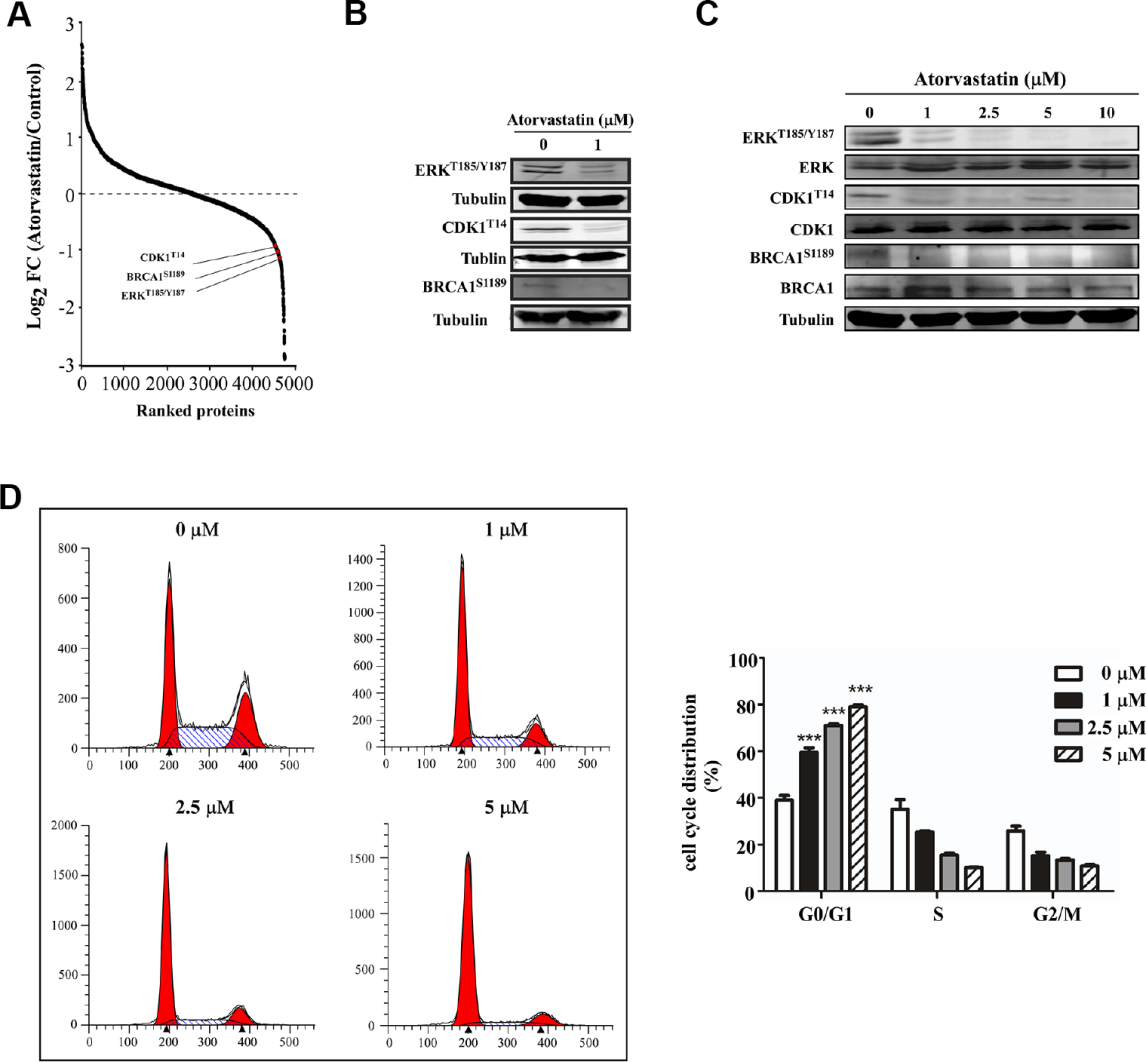
**Atorvastatin inhibits phosphorylation of ERK^T187/Y187^, CDK1^T14^, BRCA1^S1189^and induces G0/G1 arrest in ESCC cells.** (**A**) shows CDK1^T14^, ERK^T185/Y187^, and BRCA1^S1189^ are down-regulated in the phosphorylation profile. (**B**) Western blotting of atorvastatin-treated KYSE150 cells verifies the results above. (**C**) With increasing concentration of atorvastatin (0, 1, 2.5, 5, 10 and 20 μM), the levels of ERK^T185/Y187^, CDK1^T14^, and BRCA1^S1189^ are decreased. (**D**) The effects of atorvastatin on cell cycle phase were assessed in KYSE150 cells. Cells were treated with 0, 1, 2.5, and 5 μM atorvastatin and then incubated 48 h. The asterisks (* *P* < 0.05, ** *P* < 0.01, *** *P* < 0.001) indicate a significant difference between untreated control and atorvastatin-treated cells.

We analyzed phosphorylation changes upon atorvastatin treatment. 6988 phosphorylation sites were identified, among which 5809 sites were quantified (Supplementary Table 2). To evaluate statistical significance, strict criteria (t-test *P*-value < 0.05, FDR < 0.01) were applied to 3 biological replicates. Among all the quantified phosphosites, we discovered that 375 unique phosphosites were up/down regulated significantly (261 sites up, 114 sites down) according to *P*-value < 0.05 (fold-change > 1.5 or fold-change < 0.67) (Figure 4A). Next, we mapped quantified phosphosites to KEGG signal pathways (Figure 4B). Particularly, we focused on down-regulation pathways associated with cancer. According to the “String” database, we applied all background proteins in down-regulated pathways to draw PPI (protein-protein interaction), and so that significantly changed proteins would stand out (Figure 4C). Through researching the dataset, we determined phosphorylation sites for the above proteins. Down-regulated phosphosites contain ERK^T185/Y187^, CDK1^T14^, BRAC1^S1189^ (Figure 5A). Also, phosphoproteomic data indicate these phosphorylation sites were obviously down-regulated, and they were widely involved in the cancer process. Our results verified phosphoproteomic data that ERK^T185/Y187^, CDK1^T14^, BRAC1^S1189^ were strongly inhibited after atorvastatin treatment (Figure 5B). With increase of the atorvastatin dose, the phosphorylation of ERK^T185/Y187^, CDK1^T14^, and BRAC1^S1189^ were down-regulated (Figure 5C).

### Atorvastatin induces G0/G1 cell arrest of ESCC cells

Evidence demonstrated that CDK1 and BRCA1 phosphorylation was involved in cell cycle regulation [9, 10]. For example, dephosphorylation of CDK1 at T14 increases its activity and prompts G2/M progression [11]. However, BRCA1 subjected to hyperphosphorylation has been required for late G1 and S phase. Based on the literature and the phosphoproteomics dataset, we applied flow cytometry to examine whether atorvastatin affected cell cycle distribution. Results showed that atorvastatin blocked ESCC cells into G0/G1 phase (Figure 5D). With the increase of atorvastatin, the proportion of G0/G1 phase cells increased. Atorvastatin could interrupt cell cycle progression by inducing G0/G1 arrest rather than by influencing G2/M.

### Atorvastatin inhibits ESCC growth *in vivo*

**Figure 6:**
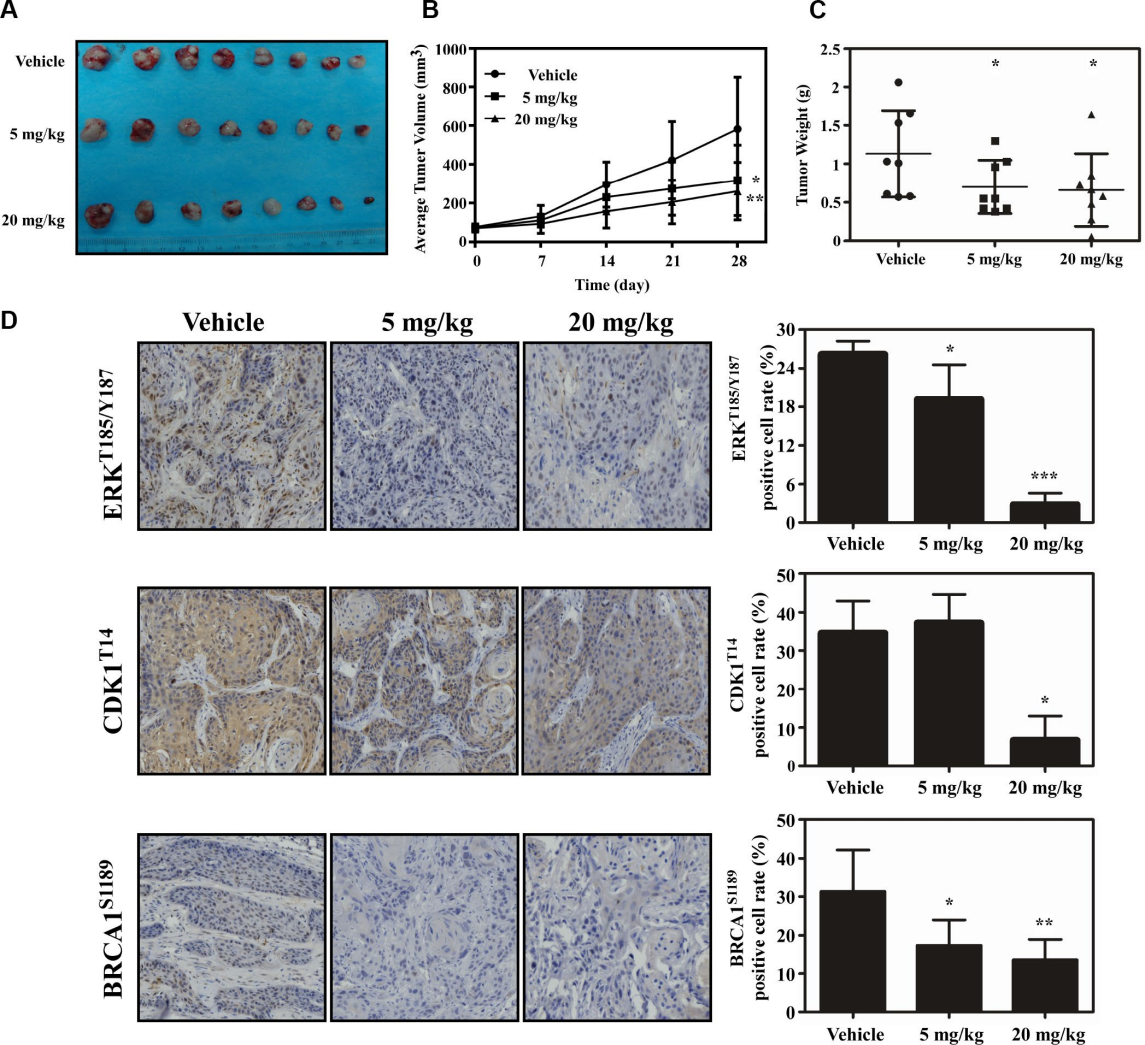
**Atorvastatin inhibits ESCC growth *in vivo*.** (**A**) The photograph shows tumors from PDX mice treated with vehicle or atorvastatin (5 mg/kg or 20mg/kg). (**B**) Tumor volume in each of the treatment groups was measured weekly, (**C**) with tumor weight measured at day 28. The asterisk (* *P* < 0.05) indicates a significant decrease in tumor volume from atorvastatin-treated mice. (**D**) The expression of ERK^T185/Y187^, CDK1^T14^ and BRCA1^S1189^ was examined by IHC (400×). Data are expressed as positive cell values ± SD. The asterisks (* *P* < 0.05, ** *P* < 0.01) indicate a significant decrease in ERK^T185/Y187^, CDK1^T14^ and BRCA1^S1189^ in atorvastatin-treated tissues compared to vehicle group.

PDX model was conducted to evaluate the anti-tumor activity of atorvastatin *in vivo*. Tumor tissue from patients was implanted into the backs of SCID mice. The mice were given atorvastatin (5 mg/kg, 20 mg/kg), or vehicle, every day over a period of 28 days. The results showed that atorvastatin significantly suppressed the volume of tumor compared with the vehicle group (Figure 6A–6B). When the mice were sacrificed, the weight of the tumor tissue was measured and the results showed that tumor weight was inhibited in mice treated with atorvastatin (Figure 6C). Additionally, mice subjected to atorvastatin treatment did not show toxicity based on the fact that body weight no loss (Supplementary Figure 1B). We then determined the findings of the phosphoproteomic profile at the tissue level by using immunohistochemistry. The results demonstrated that atorvastatin markedly decreased phosphorylation of CDK1^T14^, BRAC1^S1189^ and ERK^T185/Y187^ (Figure 6D).

## DISCUSSION

ESCC has a specifically high prevalence in Asia, especially in China [12]. Recent evidence has indicated that the incidence of ESCC has also increased in the USA [13]. Despite advances in diagnostic methods and treatment, patient prognosis has remained poor [14]. Therefore, it is important to find effective agents for ESCC treatment or prevention.

Atorvastatin, an FDA-approved drug for the treatment of dyslipidemia, was used to decrease the serum cholesterol level in clinic [15]. Myalgia is the most common adverse reaction of atorvastatin [16]. Recent studies have suggested that atorvastatin also had anti-tumor properties and these studies have mainly focused on a relationship between cholesterol biosynthesis and cancer [17]. Some plausible mechanism for atorvastatin against cancer have also been reported. By blocking byproduct of the cholesterol or mevalonate pathway including geranyl-geranyl pyrophosphate and farnesyl pyrophosphate, atorvastatin attenuated leukemic cell growth and migration [18]. Atorvastatin also induced autophagy and inhibited proliferation on prostate cancer cells through up-regulation of miR-182 [19]. Atorvastatin has been identified through modulating mitochondrial metabolism to interfere cancer process [20]. However, any other mechanism implicated in the anti-tumor effect of atorvastatin has not been clearly elucidated. In this study, we found that atorvastatin markedly decreased proliferation of ESCC cells (Figure 1A) and anchorage-independent growth (Figure 1B). We evaluated the effect of atorvastatin on ESCC growth in a PDX model (Figure 6A–6B). Results indicated that atorvastatin treatment markedly suppressed tumor growth without affecting mouse body weight. Due to the fact that the xenograft retains heterogeneity to the maximum extent, the results of the PDX model are more reliable and it is considered as the most appropriate model for preclinical study [21, 22]. Therefore, we identified atorvastatin as a prospective candidate for ESCC chemoprevention.

Several studies have recognized that atorvastatin disrupts cholesterol synthesis by blocking HMG-CoA reductase while not facilitating small G-protein activity, which is necessary for oncogenic signal pathways, especially the Ras pathway [23]. We assumed that atorvastatin may also have an unrevealed mechanism for inhibiting ESCC. Mass spectrometry is a powerful method to explore system pharmacology [24]. Here, we applied the proteome and phosphoproteome to reveal the anti-tumor mechanism of atorvastatin. In the proteome, we verified that atorvastatin down regulated the Ras signaling pathway, which was consistent with previous studies [25]. In addition, atorvastatin attenuated the cAMP signal pathway, which has been frequently observed to be aberrant in cancer cells [26, 27]. Several studies have suggested Rap1 activation was implicated in phenotype malignant tumors. In melanoma, blocking the Rap1 pathway can prevent metastasis to the lungs. Our proteomics data showed that Rap1 signal pathway were inactive upon atorvastatin treatment from KEGG analyzing. Meanwhile, multiple intracellular signal pathways related with cancer development, including MEK/ERK, PI3K/AKT, are implicated in these above pathway [27, 28].

Phosphorylation is a common post-translational modification that regulates many biological processes [29]. Evidence showed that atorvastatin decreased phosphorylation of ERK^T185/Y187^, CDK1^T14^ and BRCA1^S1189^. ERK activation is closely related to cancer development [30]. BRCA1 is a protein with various biological processes. On the one hand, mutation of BRCA1 leads to breast cancer formation. On the other hand, in response to DNA damage, BRCA1 is hyperphosphorylated by ATM [31]. BRCA1 is a modulator in cell cycle progress. The protein is differentially phosphorylated during the cell cycle. BRCA1 is dephosphorylated in early G1 while it is subjected to hyperphosphorylation in G1 late and S phases [9]. CDK1 is also a major kinase to mediate the cell cycle, and its inactivity leads to cell cycle arrest [10]. Dephosphorylation at threonine-14 residues is required for activation of CDK1 and further progression through the cell cycle [32]. Based on KEGG analysis, the proteins mapped to the cancer-related cellular signal pathway, including Th17 cell differentiation, Gap junction, Platinum drug resistance. Th17 cells are one major kind of cell in immune responses. Th17 cells and the cytokines they excrete have higher levels in tumor [33]. Connexins offer a platform for adjacent cells to communicate with each other and to transfer information. The whole exchange process is characterized by gap junction intercellular communication (GJIC). Fabien Gava found metastasis could be reduced when breast cancer cells were treated with a gap junction inhibitor [34, 35]. Furthermore, IHC reconfirmed that atorvastatin treatment markedly inhibited the phosphorylation status of ERK^T185/Y187^, CDK1^T14^ and BRCA1^S1189^.

All considered, atorvastatin suppresses the proliferation of ESCC through inhibition of the phosphorylation of ERK^T185/Y187^, CDK1^T14^ and BRCA1^S1189^ involved in multiple pathways.

## MATERIALS AND METHODS

### Cell lines

The KYSE150 and KYSE450 cell lines were grown in 1640 medium and RPMI DMEM respectively, containing 10% FBS, at 37°C in a humid incubator with 5% CO_2_. The cells were cultured and maintained for a maximum of 2 months, about 10 passages.

### Cell proliferation assay

KYSE150 and KYSE450 cells were seeded into 96 well plates, respectively at a density of 3×10^4^ and 5×10^4^ per well. After 16 h incubation, the cells were exposed to different concentration of atorvastatin (0, 1, 2.5, 5, and 10 μM) for 24, 48, 72, and 96 h. Pre-cooled PBS was used to wash the cells 3 times and the cells were fixed in 4% paraformaldehyde. Then DAPI was used to stain cell nuclei at 37°C for 20 min and then the cells were counted by machine (IN Cell Analyzer 6000).

### Soft agar assay

KYSE150 and KYSE450 cells (8×10^3^) were suspended in 6 well plates with 0.3% agar and 10% FBS at different concentrations of atorvastatin (0, 1, 2.5, 5, and 10 μM) and then cultured for 1-2 week in a 37°C incubator. Colonies were counted by IN Cell Analyzer 6000 soft agar program.

### Western blotting

The concentration of protein samples was determined using a protein assay kit (BCA Protein Assay Kit, Beyotime, China). Equal amounts of protein were resolved by SDS-PAGE and transferred onto polyvinylidene difluoride membranes (Millipore) in transfer buffer and then blocked with 5% BSA or 5% skim milk. The membranes were incubated with antibodies overnight at 4°C, and the membranes were washed 3 times with 1×TBST, followed by incubation with horseradish peroxidase (HRP) -linked appropriate secondary antibodies for 2 h at room temperature. The enhanced chemiluminescence (ECL) detection reagent was used to visualize the protein bands.

### Cell cycle analysis

KYSE150 cells were cultured in 6-well plates at density of 8×10^5^. After incubation for 24 h, the cells were given atorvastatin (0, 1, 2.5, and 5 μM) for 48 h. The cells were washed 2 times with PBS, trypsinized, again washed with pre-cooled PBS, and fixed with 1ml 70% ethanol overnight at −20°C. The cells were exposed to RNase (1 mg/kg) 5 μl for 1 h at RT, and followed by PI (10 mg/kg) 5 μl for 15 min in the dark. Cell cycle phase was measured by flow cytometry (BD Biosciences, San Jose, CA).

### PDX mouse model

Severe combined immunodeficient (SCID) mice, 6 to 8 week-old, were purchased from Vital River Labs, Beijing, China for these experiments. ESCC fragments about 1-2 mm were seeded onto the backs of the mice. When the volume of tumor reached 100 mm^3^, mice were randomly divided into 3 groups: vehicle, and atorvastatin at a dosage of 5 mg/kg or 20 mg/kg given by gavage 6 times each week. Body weight and tumor volume were measured once weekly. When the volume of the tumor mass reached about 1000 mm^3^, the mice were sacrificed and the tumor weight was measured. This study was conducted under a program approved by the Ethics Committee of Zhengzhou University (Zhengzhou, Henan, China).

### Immunohistochemical (IHC) analysis

The basic processes of IHC were followed [36]. Briefly, paraffin-embedded tissues (5 μm) were prepared for IHC analysis. The specimens were baked in a constant temperature oven 65°C for 2 h, deparaffinized, rehydrated and followed by antigen retrieval. All samples were dripped with 3% H_2_O_2_ for 10 min in dark to make endogenous peroxidases inactivate. Then slides were incubated with specific antibodies overnight at 4°C. The tissue sections were washed 3 times with 1×TBST, and hybridized with the secondary antibody for 15 min at 37°C. After DAB staining, all slides were again stained with hematoxylin, dehydrated, and covered by glass coverslips.

### Reagents and antibodies

Atorvastatin was purchased from Sigma (USA) for cell experiments. The powder was dissolved into DMSO (Sigma-Aldrich, USA) at 50 mM. Atorvastatin was purchased from Pfizer for animal experiment. ERK^T185/Y187^, CDK^T14^ were obtained from Thermo Fisher Scientific. BRCA1^S1189^ was purchased from Abcam. ERK, CDK1, and BRCA1 were obtained from Cell Signaling Technology.

### Protein extraction

KYSE150 cells were seeded into 15 cm dishes. After 16 h of culture, cells were treated with atorvastatin (1μM) for 24 h. Cells were lysed in lysis buffer with 8 M urea with 1% protease inhibitor cocktail. Samples were centrifuged to remove debris, and then the supernatant was collected.

### Trypsin digestion

5 mM dithiothreitol was added into the protein sample for 30 min at 56°C. Iodoacetamide was conducted into the samples for 15 min RT in the dark. 100 mM NH_4_HCO_3_ was applied to dilute samples to urea concentration no more than 2 M. Trypsin was added into protein samples (according to mass ratio 1:50 trypsin-to-protein) 37°C overnight, and then for 4 h digestion at a 1:100 trypsin-to-protein mass ratio.

### LC-MS/MS analysis

After the peptides were dissolved using 0.1% formic acid, samples were separated by EASY-nLC 1000 ultra-high performance liquid phase system, and then subjected to NSI source followed by Orbitrap Fusion^TM^ system analysis. Peptides were selected for MS/MS by NCE and fractions were detected in the Orbitrap at resolution of 17,500.

### Data analysis

Raw database were performed using Maxquant search engine (v.1.5.2.8) [37]. In order to facilitate database research, the results were exported into Excel [38]. The normalization of data was carried out with reference to published paper [39]. GO annotation and KEGG pathway research were conducted to analyze quantitative protein and phosphorylation sites. *P* < 0.05 was evaluated for statistical significance. FDR was set to < 1%.

### Statistical analysis

GraphPad Software 5 was used for graph creation. And data are presented as mean ± SD from more than 3 times replicates. SPSS 20.0 was applied to evaluate significant difference. Student’s *t* test or one-way ANOVA was used to determine significant difference. *P* values of <0.05 were recognized as statistically significant. * is indicated *P* values of <0.05; ** is indicated *P* values of <0.01; *** is indicated *P* values of <0.001.

## Supplementary Material

Supplementary Figure 1

Supplementary Tables

Supplementary Table 1

Supplementary Table 2
