# Supplementary material for: Proteome and phosphoproteome reveal mechanisms of action of atorvastatin against esophageal squamous cell carcinoma
Source: Aging (Albany NY). 2019 Nov 7;11(21):9530–43. doi: 10.18632/aging.102402 (PMC6874460; doi:10.18632/aging.102402)
Supplement: Supplementary Figure 1 [file aging-11-102402-s002.pdf]

SUPPLEMENTARY FIGURE

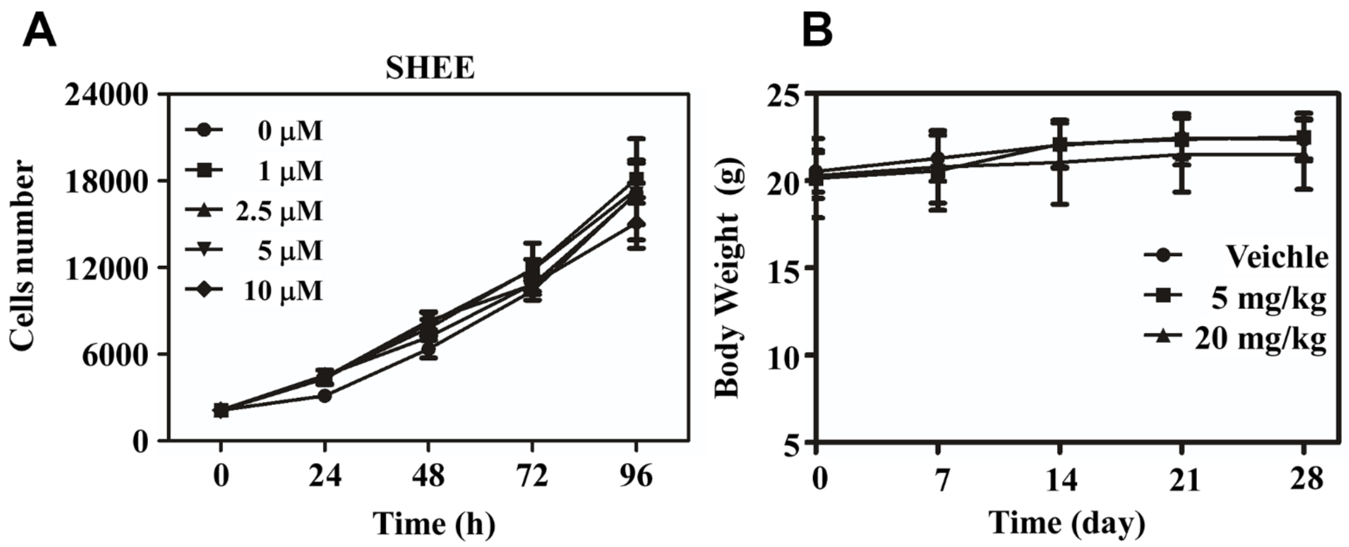

Supplementary Figure 1. (**A**) Atorvastatin has no cytotoxicity against normal esophageal epithelial cell lines, SHEE, at the same concentration. (**B**) Atorvastatin has no toxicity in mice body weight.
