# Supplementary material for: Proteome and phosphoproteome reveal mechanisms of action of atorvastatin against esophageal squamous cell carcinoma
Source: Aging (Albany NY). 2019 Nov 7;11(21):9530–43. doi: 10.18632/aging.102402 (PMC6874460; doi:10.18632/aging.102402)
Supplement: Supplementary Tables [file aging-11-102402-s001.pdf]

SUPPLEMENTARY TABLES

Please browse Full Text version to see the data of Supplementary Tables 1 and 2.

Supplementary Table 1. Summary of quantified proteins upon atorvastatin.

Supplementary Table 2. Summary of quantified phosphosites upon atorvastatin.
